# Improving Personalized Meal Planning with Large Language Models: Identifying and Decomposing Compound Ingredients

**DOI:** 10.3390/nu17091492

**Published:** 2025-04-29

**Authors:** Leon Kopitar, Leon Bedrač, Larissa J. Strath, Jiang Bian, Gregor Stiglic

**Affiliations:** 1 Faculty of Health Sciences, University of Maribor, 2000 Maribor, Slovenia; gregor.stiglic@um.si; 2 Faculty of Electrical Engineering and Computer Science, University of Maribor, 2000 Maribor, Slovenia; 3 The Nu B.V., 2333 CH Leiden, The Netherlands; leon.bedrac@gmail.com; 4 Pain Research & Intervention Center of Excellence (PRICE), University of Florida, Gainesville, FL 32608, USA; larissastrath@ufl.edu; 5 Department of Health Outcomes and Biomedical Informatics, College of Medicine, University of Florida, Gainesville, FL 32608, USA; 6 Department of Biostatistics and Health Data Science, Indiana University, Bloomington, IN 46202, USA; bianji@regenstrief.org; 7 Regenstrief Institute, 1101 W 10th St., Indianapolis, IN 46202, USA; 8 Usher Institute, University of Edinburgh, Edinburgh EH16 4UX, UK

**Keywords:** artificial intelligence, food analysis, LLM, llama, GPT, mixtral, ingredient identification, ingredient decomposition, personalized nutrition, meal customization, nutritional analysis, dietary planning

## Abstract

Background/Objectives: Identifying and decomposing compound ingredients within meal plans presents meal customization and nutritional analysis challenges. It is essential for accurately identifying and replacing problematic ingredients linked to allergies or intolerances and helping nutritional evaluation. Methods: This study explored the effectiveness of three large language models (LLMs)—GPT-4o, Llama-3 (70B), and Mixtral (8x7B), in decomposing compound ingredients into basic ingredients within meal plans. GPT-4o was used to generate 15 structured meal plans, each containing compound ingredients. Each LLM then identified and decomposed these compound items into basic ingredients. The decomposed ingredients were matched to entries in a subset of the USDA FoodData Central repository using API-based search and mapping techniques. Nutritional values were retrieved and aggregated to evaluate accuracy of decomposition. Performance was assessed through manual review by nutritionists and quantified using accuracy and F1-score. Statistical significance was tested using paired *t*-tests or Wilcoxon signed-rank tests based on normality. Results: Results showed that large models—both Llama-3 (70B) and GPT-4o—outperformed Mixtral (8x7B), achieving average F1-scores of 0.894 (95% CI: 0.84–0.95) and 0.842 (95% CI: 0.79–0.89), respectively, compared to an F1-score of 0.690 (95% CI: 0.62–0.76) from Mixtral (8x7B). Conclusions: The open-source Llama-3 (70B) model achieved the best performance, outperforming the commercial GPT-4o model, showing its superior ability to consistently break down compound ingredients into precise quantities within meal plans and illustrating its potential to enhance meal customization and nutritional analysis. These findings underscore the potential role of advanced LLMs in precision nutrition and their application in promoting healthier dietary practices tailored to individual preferences and needs.

## 1. Introduction

The incorporation of artificial intelligence (AI) across various fields has revolutionized industries such as healthcare, finance, and education, providing a high level of efficiency and accuracy. The field of nutrition is no exception. The applications of AI in nutrition include personalized nutrition, food intake tracking and recognition, nutrition monitoring, nutritional evaluation, and predictive analysis to prevent and manage diseases [1]. For example, machine learning algorithms can process individual health data, genetic profiles, and eating behaviors to generate customized nutrition recommendations, thus improving adherence to dietary guidelines and promoting better health outcomes. Santhuja et al. developed an intelligent personalized nutrition guidance system using machine learning to provide personalized dietary advice based on user-specific data [2]. Another aspect is the application of machine learning techniques by Maurya et al., who predicted chronic kidney disease and recommended appropriate diet plans for patients, addressing specific health conditions through personalized nutrition [3]. Similarly, Li et al. utilized deep learning-based near-infrared hyperspectral imaging for precise food nutrition estimation, offering innovative solutions for nutritional analysis [4]. Furthermore, Sripada et al. explored AI-driven food image classification using machine learning and deep learning to improve nutritional assessments [5]. Kim et al. employed deep learning models to classify and predict the effects of nutritional intake on obesity, hypertension, and type 2 diabetes mellitus, highlighting the role of AI in preventive healthcare [6]. These advancements demonstrate the versatility of AI in transforming personalized nutrition into a data-driven field capable of addressing diverse health challenges.

The complexity of modern food environments, driven by globalization and continuous innovation, necessitates a rigorous approach to dietary choices. Concerns about dietary habits and meal planning are growing due to the easy availability of low-nutrient foods, limited time to prepare healthy meals, and the lack of quality community-based nutrition education. As a result, many individuals find it increasingly difficult to maintain a balanced and nutritious diet. Nutrient profiling, defined as the classification of foods based on their nutritional composition to prevent disease and promote health, is a pivotal tool in this endeavor [7]. In order to do this, meal plans and foodstuffs must first be broken down to their sub-components; this is particularly time consuming for both providers and patients, and also leaves a large amount of room for human error. By leveraging artificial intelligence (AI) to aid in the deconstructing of meals into their basic ingredients, we can then perform precise quantitative and qualitative assessments of their suitability for individuals with specific health conditions and goals. This approach enables the customization of meal ingredients, enhancing the precision of dietary interventions, and underscores the growing necessity for sophisticated tools that can accurately decompose and assess the nutritional composition of diverse food combinations to adapt meals to individual needs.

Existing AI-driven approaches, such as the smartphone-based system developed by Lu et al. which employs deep neural networks to estimate the energy and macronutrient content of meals from food images, demonstrate the potential of AI in dietary analysis [8]. However, these image-based methods are limited by image quality and the inability to identify all ingredients in complex dishes. Recent developments in AI-powered dietary assessment tools have added new capabilities, such as motion sensor-based technologies that track eating events by monitoring wrist movements, jaw activity, and chewing behavior, thereby improving accuracy [9]. Additionally, hybrid methods that integrate food imagery with other data sources—like audio and depth information—have shown potential in enhancing the accuracy of nutrient estimation [10].

Large Language Models (LLMs) have shown remarkable capabilities in understanding and generating human-like text. These models, trained on extensive datasets, can comprehend and reason about complex concepts, making them well suited for a range of applications beyond basic text processing [11,12]. By leveraging the semantic understanding and contextual awareness of LLMs, it is possible to develop advanced systems for analyzing meal plans and providing personalized nutritional recommendations [8,13].

Many meal plans are composed of food items, usually listed as their final product (i.e., hummus) rather than their ingredient list. Ingredients can be basic, unable to be decomposed further, or compound, which are themselves the product of more than one ingredient.

Analyzing the nutritional content of meals—known as nutrient profiling—requires breaking down compound ingredients into their individual components. Here are several reasons why this is essential. Firstly, it enables detection of particular ingredients that could pose problems for individuals with food allergies or intolerances. Dissecting compound ingredients helps to identify and substitute problematic ingredients. Secondly, this approach allows for higher precision in the evaluation of the nutritional profile of the meal to ensure it is balanced and healthy, rather than attempting to assess an entire meal plan containing compound ingredients. In connection with ingredient databases, it can also be used to estimate the cost of specific meals. This can be especially important in cases where a large number of meals are prepared. Furthermore, this process offers personalization and customization of meal plans to individual preferences, dietary needs, and health goals. Additionally, a structured framework for food group-based evaluation, aligned with nutritional guidelines, is essential for implementing effective LLMs into meal planning. Such models consider both the harmful overconsumption of nutrients, such as energy, total fat, saturated fatty acids, trans fatty acids, free sugars, and sodium, and the underconsumption of essential nutrients, including vitamins and minerals.

The application of these models extends beyond individual health benefits; they hold significant potential for integration into institutional settings such as schools, kindergartens, and canteens. By promoting healthier food choices and addressing both nutrient excesses and deficiencies, LLM-aided nutrient profiling can play a critical role in combating diet-related noncommunicable diseases and fostering overall public health.

Lu et al. tackle the composition problem using a smartphone-based system that can estimate the energy and macronutrient (carbohydrate, protein, fat) content of meals based on food images captured by the user. The system utilizes deep neural networks, specifically, a segmentation approach with the Mask R-CNN [14] framework, to detect, segment, and recognize the foods in the images [8].

Building on findings from Osowiecka et al., which highlight factors contributing to dysbiosis and dysbiosis-related health problems in individuals with excessive body mass [15], personalized meal planning that emphasizes nutrient-dense, fiber-rich foods and limits processed ingredients can play a critical role in preventing excessive body mass by supporting a healthier gut microbiota and reducing the risk of dysbiosis-related metabolic disorders. The study also found that individuals with a BMI ≥ 25 kg/m^2^, indicative of pre-obesity and obesity, demonstrated a higher prevalence of factors associated with dysbiosis compared to those with a normal BMI.

Our paper explores the potential of using LLMs to segment compound ingredients into simple ingredients and map these items to the United States Department of Agriculture (USDA) FoodData Central repository. By leveraging the advanced capabilities of these LLMs, we aim to evaluate their ability to analyze complex meal plans and provide detailed nutritional breakdowns and assessments. In comparison to the approach used by Lu et al. [8], our approach does not focus in detail on calorie and macronutrient or micronutrient content, nor does it estimate these factors. These aspects will be explored in future studies. Our approach relies only on the name of the meal, which enables its application to a wider range of applications where only a short description or the name of the meal is available.

## 2. Materials and Methods

### 2.1. Study Design

Our study employed primarily a quantitative approach, supplemented by a qualitative approach, to compare and investigate the ability of different LLMs to breakdown and assess nutritional values using the USDA FoodData repository for reference. The rationale behind choosing a quantitative approach was to allow for rigorous measurement and statistical analysis of variables, enabling us to draw objective conclusions about the impact of LLMs’ reasoning on compound ingredient, meal plan decomposition.

We compared three different LLMs: GPT-4o [16] and two open-sourced models Llama-3 (70B) [17], and Mixtral (8x7B) [18]. Models were compared in terms of their ability to identify and decompose compound ingredients into basic components. These basic components or ingredients were then mapped to results from the USDA FoodData Central repository.

Mapping basic ingredients to USDA FoodData entries allowed us to obtain a quantitative overview of the nutrient composition of compound ingredients. Performance of the decomposition into basic ingredients was also manually assessed.

### 2.2. Data Analysis Tools and Techniques

The research was conducted using the Python programming (version 3.12.8) language and its corresponding libraries, with OpenAI [19] and Perplexity [20] at the forefront.

The OpenAI library, specifically GPT-4o, was used for generating meal plans and mapping extracted basic ingredients to the entries of USDA FoodData repository. We chose one model for that task since meal generation and mapping is not the primary goal of this study.

Both LLM’s related libraries were used for compound ingredient identification and its decomposition to basic ingredients.

We utilized the USDA FoodData Central API to retrieve all detailed data related to food and food composition [21]. The USDA API offers access to the Standard Reference (SR) Legacy data (last release on April 2018), USDA Global Branded Foods Database (updated monthly), Foundation Foods (updated twice a year, in April and October), Food and Nutrient Database for Dietary Studies (FNDDS) (updated every two years), and experimental foods (updated twice a year, in April and October).

### 2.3. Statistical Analysis

All statistical analyses were performed using Python 3.12.8, with the pandas library for data preprocessing, scipy.stats for hypothesis testing, and statsmodels for statistical modeling. Accuracy and F1-scores were calculated for each model across a shared evaluation dataset, and results were supported with 95% confidence intervals. Accuracy reflects the ratio of correct predictions to the total number of samples in the test set [22]. On the other hand, the F1-score combines precision and recall using their harmonic mean, ensuring that neither is disproportionately favored. It produces values between 0 and 1, where 1 reflects ideal precision and recall [22].

To assess the statistical significance of performance differences between models, we first evaluated the normality of the accuracy distributions using the Shapiro–Wilk test. Based on the results, either paired *t*-tests (for normally distributed data) or Wilcoxon signed-rank tests (for non-normal distributions) were applied for pairwise comparisons. A significance threshold of α=0.05 was adopted throughout the study.

### 2.4. Large Language Models

In this study, we compared three distinct Large Language Models (LLMs): GPT-4o [16], Llama-3 (70B) [17], and Mixtral (8x7B) [18]. These models were selected to represent a spectrum of capabilities and accessibilities within the LLM landscape.

GPT-4o is a generative pre-trained transformer (GPT) model that is a step closer to a more natural human–computer interaction by accepting and generating a wide range of sources, including text, audio, images, and video. In comparison to its predecesor, GPT-4 Turbo, it provides a similar level of performance on English text and code-related tasks. GPT-4o specifically offers superior vision and audio understanding capabilities, at a 50% lower cost, opening up new possibilities for multimodal applications [16]

Llama-3 (70B) is an open-sourced LLM developed by Meta AI. At present, it stands as the most advanced openly available LLM, outperforming numerous proprietary models in widely recognized industry tests [17].

Mixtral (8x7B) is a Sparse Mixture of Experts (SMoE) language model. Architecturally, it shares the same framework as Mistral 7B, except each layer incorporates eight feedforward blocks. Each token routes through two feedforward blocks selected by a router network, and combines their outputs. Consequently, each token has access to 47 billion parameters, but only 13 billion parameters are active during inference. In terms of performance, it surpasses GPT-3.5 Turbo, Claude-2.1, Gemini Pro 1.0, and Llama-2 (70B) on the chat model on human benchmarks [18].

### 2.5. Methodology

The research process involved several key steps: generation of meal plans, decomposition of compound ingredients, USDA FoodData subset creation, mapping to USDA FoodData, nutrient composition calculation, aggregation of nutrient data, and the evaluation process.

#### 2.5.1. Generation of Meal Plans:

Using GPT-4o with default parameters, 15 meal plans were generated across five iterations. Of these 15 plans, five were designated for breakfast, five for lunch, and five for dinner. Each plan included precise portion sizes and specific quantities or weights of individual ingredients. Meal plans were generated independently. The prompt used for generating the meal plans is detailed in Prompt 1.
**Prompt 1** Meal generation (Prompt)Create a detailed one-day <INPUT_meal_type> plan. Ensure the meal plan includes precise portion sizes, specific quantities or weights of individual ingredients, and diverse cooking methods. Include compound ingredients like Chicken Cacciatore, Vegetable Ratatouille, or similar. However, avoid including ingredients included in the following list: <INPUT_list>, Structure the results in a formatted HTML format similar to the provided example: <INPUT_html_format>.

Where <INPUT_meal_type> holds one of three possible meal types: breakfast, lunch, or dinner. Another input, <INPUT_list>, begins as an empty list but gradually accumulates ingredients each time a meal plan is generated. This was implemented with the intention of ensuring diversity and uniqueness in the selection of compound ingredients, thereby thoroughly testing the LLMs. The final input, <INPUT_html_format>, accepts a predefined HTML format for displaying meal plans in a clear and readable manner. The HTML table format includes ingredient names, quantities (cups, slices), and portion sizes (grams, deciliters). The limited number of columns ensures that LLMs are evaluated primarily on their ability to identify compound ingredients, break them down, and reasonably distribute portion sizes among basic ingredients.

#### 2.5.2. Decomposition of Compound Ingredients:

For each model (GPT-4o, Llama-3 (70B), Mixtral (8x7B)), we analyzed 15 meal plans and tasked the models with identifying and deconstructing compound ingredients into their simpler components, the basic ingredients. For example, ‘Chicken Marsala’ is a compound ingredient that can be broken down into basic ingredients, such as chicken breast, Marsala wine, olive oil, garlic, mushrooms, chicken broth, flour, salt, and black pepper. The process was conducted using the following prompt as presented in Prompt 2.
**Prompt 2** Identification and decomposition of the compound ingredients. (Prompt)Analyze the meal plan provided in an HTML table (enclosed within <table> and </table> tags): <INPUT_meal_plan>. Identify compound ingredients listed under the ‘Ingredient’ column that consist of multiple basic ingredients. Use the ‘Ingredient Details’ column for additional information. For each identified compound ingredient, report the names of these items along with their specific ingredients and estimated quantities based on the ‘Portion Size (g, dL)’ column, formatted in a dictionary of dictionaries. Ensure all ingredients are specific (e.g., ‘carrots’, ‘spinach’, etc., instead of ‘vegetables’). If items can be further broken down, do it. Example format: ‘Complex Food Item 1’: ‘Ingredient 1’: ‘50 g’, ‘Ingredient 2’: ‘100 g’, “Complex Food Item 2”: ‘Ingredient 1’: ‘200 g’, ‘Ingredient 2’: ‘150 g’. Return only the formatted dictionary encapsulated by dollar signs ($Content$). Dictionary should not contain any comments. If no complex food items meeting the criteria are identified, return an empty dictionary. Ensure that the quantities of each ingredient accurately reflect the specified portion size within each compound ingredient as detailed in the meal plan. For ingredients where the exact quantity cannot be determined, provide a reasonable estimate instead of leaving it unspecified.

Where <INPUT_meal_plan> is the output of Prompt 1.

#### 2.5.3. USDA FoodData Subset Creation

Each basic ingredient derived from a compound ingredient contributes to a refined subset of USDA FoodData. This subset is temporarily stored and utilized during the acquisition process of nutritional content for the basic ingredient. The subset is generated by Fooddata Central’s API function ‘search(apikey,‘basic ingredient’)’. This subset initially includes only entries of type ‘SR Legacy’ or ‘Foundation Foods’. In the event of insufficient entries, entries from the ‘Survey type (FNDDS)’ are included. These three data types originate from the USDA. If entries are still not available, attempts are made to include types ‘Experimental Foods’ and ‘Branded Foods’. The subset includes only the description name and FoodData Central ID (fdcId). We intentionally excluded nutritional content for each entry to allow the model to make decisions based solely on the description name.

#### 2.5.4. Mapping to USDA FoodData

Each basic ingredient was then mapped to the most appropriate entry in the narrowed selection of USDA FoodData Central repository entries. Narrowed selection is determined using GPT-4o. The specific prompt for mapping is shown in Prompt 3.
**Prompt 3** Mapping the ingredient to FoodData Central’s list of ingredients (Prompt)You are tasked with matching a given ingredient <food_item> to the most appropriate one from the following list of ingredients: <list_of_food_items>. Each ingredient possesses unique characteristics that influence its compatibility with others. Your objective is to identify which ingredient from the list best complements the given one, considering factors such as flavor profile, cooking methods, and culinary traditions. Return only the ‘fdcId’ value of the best match encapsulated in dollar signs (e.g., $21341$). Do not include any comments.

#### 2.5.5. Nutrient Composition Calculation

Once the ingredient is matched with the USDA FoodData entry and its ID number is obtained, we proceed with fetching the nutritional composition of the matched entry. The fetching of nutrient composition is performed by the FoodData Central API function ‘nutrients(apikey, fdcId)’, which accepts an API key and the ID of the ingredient. The nutritional composition is then further adjusted to correspond to the actual proposed weight of the ingredient, as FoodData provides compositions based on 100 g (or 100 mL) of the ingredient.

#### 2.5.6. Aggregation of Nutrient Data

After retrieving the nutrient composition of all basic ingredients within a compound ingredient, the composition is aggregated for each compound ingredient, representing its total nutritional value. The nutrient values were aggregated to determine the overall nutrient composition of the compound ingredient. This provided a detailed breakdown of compound ingredients and gives insights into nutrients such as total protein, fats, carbohydrates, vitamins, and minerals.

#### 2.5.7. Evaluation Process

The evaluation of LLMs focuses on their ability to accurately identify compound ingredients and decompose these ingredients into basic components.

- Identification by Nutritionists: Nutritionists review 15 meal plans each to identify compound ingredients. These identifications serve as ground truth positives.
- Comparison with Model Predictions: The compound ingredients identified by the nutritionists are compared with those predicted by the model. This comparison is used to calculate accuracy and F1-score. Accuracy measures the proportion of correctly identified compound ingredients out of the total number of ingredients. F1-score provides a balance between precision and recall, offering a comprehensive measure especially useful in cases of class imbalance.
- Quality control: The nutritionists assess the decomposed basic ingredients and their quantities to confirm that they are realistic and consistent with the compound ingredients.

To ensure consistency and reliability in the manual assessment of decomposition results, nutritionists were first provided with clear instructions detailing the criteria for evaluating the accuracy of ingredient decomposition, including specific examples and edge cases.

Statistical significance in performance comparisons between models is determined using either a paired *t*-test or a Wilcoxon signed-rank test, based on the normality of the data as indicated by the Shapiro–Wilk test.

## 3. Results

### 3.1. Summary of Compound Ingredients in the Meal Plans

The 15 meals consisted of a total of 101 ingredients, among which ‘Milk (or plant-based milk)’ and ‘egg/s’ appeared in two meal plans, resulting in 99 unique ingredients. The ingredient ‘Mixed Green Salad’ appeared in four different variations: ‘Mixed Green Salad (Romaine, Cucumber, Carrot)’, ‘Mixed Greens Salad’, ‘Mixed Greens Salad (with lemon vinaigrette)’, and ‘Mixed Greens Salad (without lemon vinaigrette)’.

Out of these 99 distinguishable ingredients, GPT-4o predicted 40 to be compound ingredients, while Llama-3 (70B) predicted 51 and Mixtral (8x7B) predicted 49 compound ingredients.

There were 31 common compound ingredients predicted by all three models, including the four variations of mixed green salad that were mentioned earlier. The pool of ingredients is slightly imbalanced, with a greater representation of basic ingredients (68%) over compound ingredients (32%).

### 3.2. Identification of Compound Ingredients

Overall performance based on accuracy (ACC) (Table 1) and F1-score (Table 2) shows that Llama-3 (70B) performs with higher average accuracy and F1-score (ACC: 0.893 (95% CI: 0.85–0.94); F1-score: 0.894 (95% CI: 0.84–0.95)), than GPT-4o (ACC: 0.835 (95% CI: 0.78–0.89); F1-score: 0.842 (95% CI: 0.79–0.89)) and Mixtral (8x7B) (ACC: 0.666 (95% CI: 0.59–0.75); F1-score: 0.69 (95% CI: 0.62–0.76)).

Results from the first human evaluator show significant differences between GPT-4o and Mixtral (8x7B) (t(14) = 2.75, *p* < 0.05), and between Llama-3 (70B) and Mixtral (8x7B) (t(14) = 3.29, *p* < 0.01), indicating that Mixtral (8x7B) performs significantly worse in terms of accuracy compared to both GPT-4o and Llama-3 (70B). However, there was no significant difference between GPT-4o and Llama-3 (70B) in accuracy (V = 12.5, *p* = 0.48). Similar observations were made in terms of F1-score (GPT-4o and Mixtral (8x7B) (t(14) = 3.13, *p* < 0.01); GPT-4o and Llama-3 (70B) (V = 11, *p* = 0.36); Llama-3 (70B) and Mixtral (8x7B) (t(14) = 3.1, *p* < 0.01)).

Results from the second evaluator show significant differences only between Llama-3 (70B) and Mixtral (8x7B) (V = 0.87, *p* < 0.05) in terms of accuracy, while there were no differences between other pairs (GPT-4o and Llama-3 (70B) (t(14) = −1.37, *p* = 0.19); GPT-4o and Mixtral (8x7B) (t(14) = 1.76, *p* = 0.1)). Similar observations were made in terms of F1-score (GPT-4o and Mixtral (8x7B) (t(14) = 1.45, *p* = 0.17); GPT-4o and Llama-3 (70B) (t(14) = −1.45, *p* = 0.17); Llama-3 (70B) and Mixtral (8x7B) (t(14) = 2.63, *p* < 0.05)). This shows that Llama-3 (70B) performed significantly better than Mixtral (8x7B).

The third evaluator’s results show significant differences between GPT-4o and Mixtral (8x7B) (t(14) = 3.56, *p* < 0.01), and between Llama-3 (70B) and Mixtral (8x7B) (V = 55, *p* < 0.01), indicating that Mixtral (8x7B) performs significantly worse in terms of accuracy compared to both remaining models. Similarly to Evaluator 1’s results, there was no significant difference between GPT-4o and Llama-3 (70B) in accuracy (V = 14, *p* = 0.62). Similar observations were made in terms of F1-score (GPT-4o and Mixtral (8x7B) (t(14) = 3.19, *p* < 0.01); GPT-4o and Llama-3 (70B) (t(14) = −0.95, *p* = 0.36); Llama-3 (70B) and Mixtral (8x7B) (t(14) = 3.7, *p* < 0.01)).

Pooling the results from all three evaluators, there are no significant differences between GPT-4o and Llama-3 (70B) (V = 105, *p* = 0.12), while Mixtral (8x7B) performance is significantly worse than both models in both metrics.

### 3.3. Decomposition of Compound Ingredients

GPT-4 returned salt, sugar, or pepper only three times as a result of the decomposition of compound ingredients. In these instances, it provided 1 g of salt twice and 1 g of pepper once, which amounts to 2–3 pinches considering that one pinch weighs approximately 0.25 g.

Llama-3 (70B), on the other hand, included these basic ingredients only twice. Once, it added 10 g of sugar and 3 g of salt to a 58-gram wholegrain English muffin. In comparison, Mixtral (8x7B) seasoned the muffin with only 2 g of sugar and 3 g of salt, making it more savory than sweet.

Mixtral (8x7B) included these ingredients more often, a total of eight times, with specific quantities provided four times. These quantities ranged from 1–3 g of salt and 1–2 g of pepper. Notably, Chicken Marsala (200 g), Chicken Cacciatore (240 g), and Vegetable Ratatouille (200 g) contained 2 g, 1 g, and 3 g of salt, respectively. Chicken Marsala, according to Llama-3 (70B), was more seasoned with 5 g of salt.

**Figure 1:**
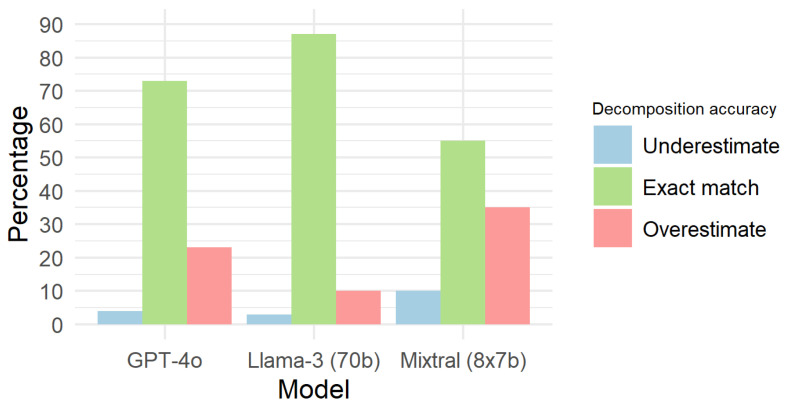
Accuracy decomposition across models.

Figure 1 illustrates the performance of different models in matching the weight of compound ingredients with their basic components. Llama-3 (70B) provided the sum of basic ingredients exactly matching the weight of the compound ingredient 87% of the time and exceeded the weight in 10% of cases. GPT-4o matched the weight 73% of the time and exceeded it in 23% of cases. Mixtral (8x7B) matched the weight 55% of the time and exceeded it in 35% of cases.

Table 3 presents a list of compound ingredients whose quantities were overestimated by 25% of the actual weight. Among these, GPT-4o and Mixtral (8x7B) estimated five (5) instances where the total weight was one quarter greater than the proposed compound ingredient’s base weight, whereas Llama-3 (70B) estimated this in only three (3) cases. Interestingly, Llama-3 (70B) only overdosed on compound ingredients that were also overdosed by the other two models.

**Figure 2:**
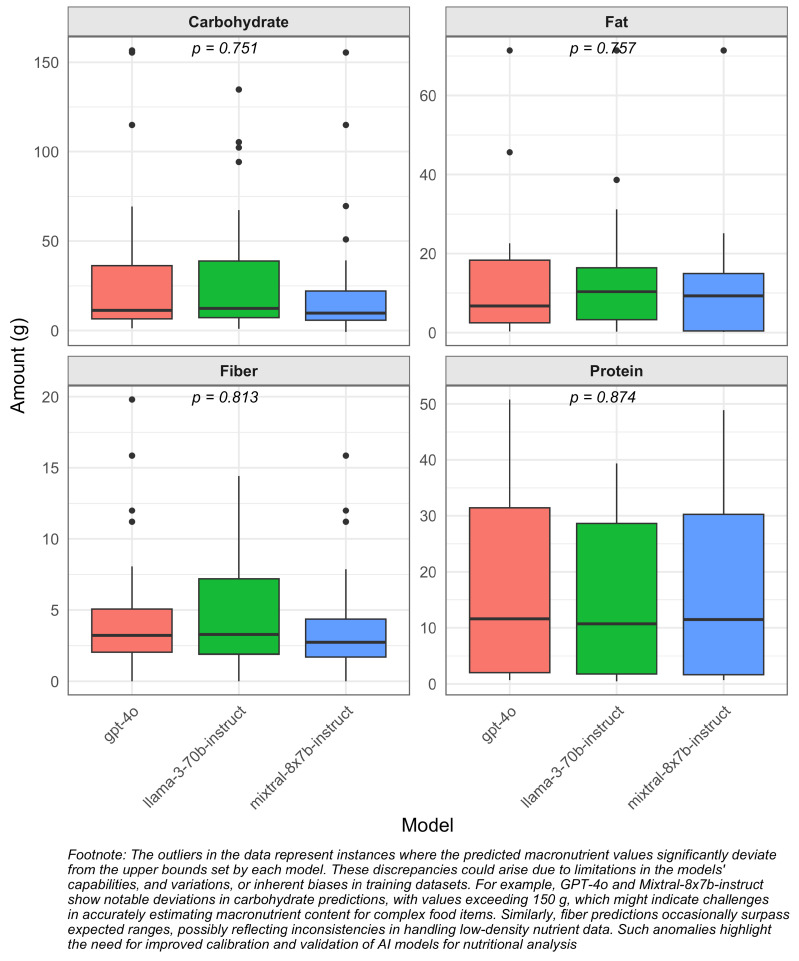
Macronutrient estimates across models, with outliers indicated by black dots.

Figure 2 provides a comparison of macronutrient estimates across three models: Mixtral (8x7B), Llama-3 (70B), and GPT-4o. The results for each model are as follows: For protein, Mixtral (8x7B) estimates 11.47 g (IQR: 1.65–30.24), Llama-3 (70B) reports 10.71 g (IQR: 1.77–28.63), and GPT-4o estimates 11.60 g (IQR: 2.01–31.45). In terms of fat, Mixtral (8x7B) provides an estimate of 9.28 g (IQR: 0.43–14.97), Llama-3 (70B) reports 10.36 g (IQR: 3.27–16.42), and GPT-4o estimates 6.73 g (IQR: 2.48–18.34). For carbohydrates, Mixtral (8x7B) reports 9.71 g (IQR: 5.74–22.16), Llama-3 (70B) estimates 12.34 g (IQR: 7.18–38.88), and GPT-4o estimates 11.25 g (IQR: 6.51–36.29). Regarding dietary fiber, Mixtral (8x7B) provides an estimate of 2.74 g (IQR: 1.70–4.36), Llama-3 (70B) reports 3.28 g (IQR: 1.91–7.20), and GPT-4o estimates 3.22 g (IQR: 2.04–5.06).

The evaluation of GPT-4o, Llama-3 (70B), and Mixtral (8x7B) models in decomposing compound ingredients revealed varied accuracy across different meal types and iterations. Common issues included the exclusion of oils, seasonings, and sweeteners, particularly in compound ingredients like scrambled eggs, mixed green salads, and ratatouille. While GPT-4o often missed decomposing certain ingredients like wholewheat toast and herb-roasted salmon, Llama-3 (70B) demonstrated a slightly better culinary accuracy, such as adding olive oil to ratatouille. Mixtral (8x7B), on the other hand, struggled with breaking down components of hummus and certain dressings but provided accurate decompositions in simpler items. Overall, all models consistently struggled with accurately identifying and integrating seasonings and cooking methods, resulting in incomplete nutritional profiles.

## 4. Discussion

Mixtral (8x7B) consistently underperforms in accuracy and F1-score compared to both GPT-4o and Llama-3 (70B) across all evaluators, with significant discrepancies found in several comparisons. Conversely, GPT-4o and Llama-3 (70B) generally exhibit similar performance levels, as no consistent significant differences were detected between them. The dataset size was limited to 15 meal plans, partly due to the significant cost and effort required for manual validation by nutritionists, which constrained the scope of the study. This study’s results are consistent with earlier research on AI-generated meal planning. Tools like Gemini and ChatGPT (3.5) have shown they can fulfill dietary reference intakes (DRIs) for both macro- and micronutrients across different eating patterns. However, careful consideration is necessary when applying these tools to restrictive diets, as they may lead to deficiencies in nutrients such as vitamin D and fluoride [23]. Similarly, ChatGPT-4 has shown potential to produce personalized weight-loss diet plans comparable in effectiveness and flexibility to those offered by clinical nutrition experts, with only five out of 14 experts able to distinguish the AI-generated plan [24].

None of the three models included basic ingredients such as salt, pepper, and sugar frequently enough. However, Mixtral (8x7B) tends to include basic ingredients such as sugar, pepper, and salt more frequently compared to GPT-4o and Llama-3 (70B). Specifically, Mixtral (8x7B) provided the quantity of these ingredients in 12.9% of the cases evaluated, whereas GPT-4o did so in 9.7% and Llama-3 (70B) in 6.5%. It appears, that Mixtral (8x7B) has learned to prioritize these basic ingredients more consistently when generating recipes or related text compared to the other models tested.

The analysis highlights the strengths and limitations of the GPT-4o, Llama-3 (70B), and Mixtral (8x7B) models in the context of meal decomposition. While all models showed potential in identifying basic ingredients, their inconsistent handling of oils, seasonings, and complex food items points to areas needing improvement. Future research could incorporate domain-specific reference data for better handling of seasonings and oils, ensuring more accurate and context-aware ingredient decomposition. Llama-3 (70B)’s better performance in certain culinary details suggests it might be more suitable for applications requiring detailed ingredient decomposition. Additionally, testing on other cuisines would validate the models’ adaptability to diverse meal plans, as ingredient naming conventions and combinations differ significantly. Expanding the dataset to include a wider range of cultural cuisines could reveal potential biases or gaps in ingredient identification and improve the generalizability of these models. Future improvements should focus on refining these aspects to provide precise and reliable nutritional analysis, ultimately aiding users in better understanding the nutritional composition of their meals. To enhance decomposition accuracy, future work could explore ensemble methods combining multiple LLMs or incorporating feedback loops for iterative improvement.

Providing a larger amount of basic ingredients than what remains in the final cooked dish does not necessarily indicate an error by the model. Ingredients commonly lose weight during food preparation due to liquid evaporation, particularly when using thermal cooking techniques. For instance, GPT-4o recommended using 10 g of butter and 20 g of milk for ‘Scrambled eggs’. Although this may appear excessive, given that scrambled eggs cook relatively quickly, it is essential to recognize that some ingredient loss occurs during cooking. However, precision becomes crucial in personalized nutrition, especially when considering excesses of fat or carbohydrates, as even minor changes in ingredient quantities can significantly alter the nutritional profile and energy density [25]. For example, in the case of a ‘Grilled Vegetable Frittata’, the total recommended ingredients exceeded the final compound ingredient weight by 162 g across all models. This included ‘Eggs’ (100 g), ‘Bell Peppers’ (75 g), ‘Onions’ (40 g), ‘Zucchini’ (65 g), ‘Cheddar Cheese’ (28 g), and ‘Herbs (parsley, basil)’ (4 g).

The analysis of macronutrient estimates across the models Mixtral (8x7B), Llama-3 (70B), and GPT-4o reveals that, despite some variations, the overall estimates are generally consistent. The interquartile ranges (IQRs) for each nutrient overlap significantly among the models, suggesting that the observed differences are minor and do not indicate substantial discrepancies in the breakdown of compound ingredients. The close alignment in protein estimates across the models suggests a robust agreement in their ability to analyze protein content. Similarly, while there are some variations in fat and carbohydrate estimates, the overlapping IQRs indicate that these difference are not significant. This is further supported by the ANOVA results (fat: F = 0.158, *p* = 0.854; carbohydrates: F = 0.197, *p* = 0.822). The dietary fiber estimates also show a high degree of consistency among the models. These findings imply that all three models provide reliable estimates for macronutrient content, with no significant deviations in their performance. This consistency reinforces the models’ effectiveness in macronutrient analysis and supports their use in various applications where accurate nutritional information is crucial. However, food additives associated with health risks may not be accurately represented in the USDA data, highlighting a limitation of LLMs in conducting nutritional analyses using basic ingredients.

The insights gained from this study can have significant real-world applications. For instance, integrating ingredient decomposition into dietary planning tools can enhance personalized nutrition recommendations, making them more accurate and tailored to individual dietary needs. Such applications could benefit from the model’s ability to provide precise ingredient quantities and composition, which are crucial for developing detailed nutritional profiles. Additionally, leveraging this research can help refine health apps that offer dietary guidance, thereby improving their utility in managing specific health conditions or achieving dietary goals. Exploring these applications in future work could close the gap between theoretical findings and practical use, offering meaningful benefits to users.

The decision to prioritize ‘SR Legacy’ and ‘Foundation Foods’ over ‘Experimental Foods’ and ‘Branded Foods’ is grounded in several considerations. While the ‘Experimental Foods’ are based on scientific publications, with entries being rare, the ‘Branded Foods’ are included in abundance (e.g., 7928 branded entries for ingredient ‘blueberries’ in comparison to two for experimental foods). Additionally, approximately 100 entries share an identical description name, posing challenges for LLM to differentiate based solely on this criterion. Furthermore, adding other information, such as food category, brand, brand owner, and market country (only two distinctive countries) do not provide distinguishing value.

The prompt (see Prompt 3) requests the FoodData ID due to the previously mentioned issue of identical description names, thereby bypassing reliance on description names. Additionally, the prompt suggests enclosing the answer in dollar signs to ensure responses avoid unnecessary comments, despite explicitly discouraging them.

When mapping basic ingredients to USDA data, one may encounter issues related to API limits. The FoodData API limits the number of requests to 1000 per hour per IP address, equivalent to one request every 3.6 s [26]. Exceeding this limit results in a temporary block for one hour. Our research was not particularly demanding. The FoodData API calls were not executed continuously for an hour, they were idle while the model processed the retrieval of compound ingredient identifications from the meal plan before providing answers. Therefore, the required wait time after each FoodData API request easily elapsed. As a result, while these limitations caused the analysis to take slightly longer, they did not affect the retrieval of data or impact any results.

## 5. Conclusions

Our study highlights the capabilities of LLMs in significantly enhancing meal planning by accurately identifying compound ingredients. These models demonstrate a strong proficiency in recognizing complex ingredient combinations, contributing to more efficient and informed meal preparation. However, despite their strengths, LLMs fall short in the precise decomposition of compound ingredients into their individual components. This limitation suggests that while LLMs can greatly enhance meal planning, there remains room for improvement in their ability to break down and analyze compound ingredient content.

## Figures and Tables

**Table 1:** Overall average performance (with 95% CI) based on accuracy with *p*-values for comparisons between models.

Evaluator	GPT-4o	Llama-3 (70B)	Mixtral (8x7B)	*p*-Value (GPT-4o vs. Llama-3)	*p*-Value (GPT-4o vs. Mixtral)	*p*-Value (Llama-3 vs. Mixtral)
1	0.838 (0.73–0.95)	**0.885** (0.79–0.98)	0.566 (0.39–0.74)	0.48	<0.05	<0.01
2	0.806 (0.71–0.91)	**0.889** (0.83–0.95)	0.729 (0.62–0.84)	0.19	0.1	<0.05
3	0.862 (0.77–0.95)	**0.906** (0.86–0.96)	0.701 (0.6–0.8)	0.62	<0.01	<0.01
Overall	0.835 (0.78–0.89)	**0.893** (0.85–0.94)	0.666 (0.59–0.75)	0.12	<0.05	<0.05

The highest average performance is highlighted in bold. *p*-values represent the significance of the pairwise comparisons between models.

**Table 2:** Overall average performance (with 95% CI) based on F1-score with *p*-values for comparisons between models.

Evaluator	GPT-4o	Llama-3 (70B)	Mixtral (8x7B)	*p*-Value (GPT-4o vs. Llama-3)	*p*-Value (GPT-4o vs. Mixtral)	*p*-Value (Llama-3 vs. Mixtral)
1	0.842 (0.75–0.94)	**0.875** (0.74–1.01)	0.612 (0.48–0.74)	0.36	<0.01	<0.01
2	0.824 (0.74–0.91)	**0.902** (0.85–0.96)	0.751 (0.64–0.86)	0.17	0.17	<0.05
3	0.861 (0.78–0.94)	**0.904** (0.88–0.93)	0.708 (0.65–0.76)	0.36	<0.01	<0.01
Overall	0.842 (0.79–0.89)	**0.894** (0.84–0.95)	0.690 (0.62–0.76)	0.14	<0.05	<0.05

The highest average performance is highlighted in bold. *p*-values represent the significance of the pairwise comparisons between models.

**Table 3:** Compound ingredients whose content was overestimated by 1/4 of the base weight.

Model	Compound Ingredients
GPT-4o	‘Scrambled Eggs’ **‘Oatmeal with Fresh Fruit’** **‘Grilled Vegetable Frittata’** **‘Whole Grain Pancakes’** ‘Steel-Cut Oatmeal’
Llama-3 (70B)	**‘Oatmeal with Fresh Fruit’** **‘Grilled Vegetable Frittata’** **‘Whole Grain Pancakes’**
Mixtral (8x7B)	**‘Oatmeal with Fresh Fruit’** **‘Grilled Vegetable Frittata’** **‘Whole Grain Pancakes’** ‘Whole Wheat Bagel with Light Cream Cheese’ ‘Chicken Marsala’

Common compound ingredients are highlighted in bold.

## Data Availability

The data presented in this study are available in OSF at https://osf.io/sb84r/files/.

## Abbreviations

The following abbreviations are used in this manuscript:

ACC	accuracy
AI	artificial intelligence
CI	confidence interval
CNN	convolutional neural network
FNDDS	food and nutrient database for dietary studies
GPT	generative pre-trained transformer
HTML	hypertext markup language
IP	Internet Protocol
IQR	interquartile range
LLM	large language model
SMoE	sparse mixture of experts
USDA	U.S. Department of Agriculture
